# Absolute Measurement of Material Nonlinear Parameters Using Noncontact Air-Coupled Reception

**DOI:** 10.3390/ma14020244

**Published:** 2021-01-06

**Authors:** Hyunjo Jeong, Sungjong Cho, Shuzeng Zhang, Xiongbing Li

**Affiliations:** 1 Department of Mechanical Engineering, Wonkwang University, Iksan, Jeonbuk 54538, Korea; 2 Nondestructive Testing (NDT) Research Center, Seoul National University of Science and Technology, Seoul 01811, Korea; cho-sungjong@seoultech.ac.kr; 3 School of Traffic and Transportation Engineering, Central South University, Changsha 410075, China; sz_zhang@csu.edu.cn (S.Z.); lixb213@csu.edu.cn (X.L.)

**Keywords:** nonlinear parameter, noncontact reception, air-coupled receiver, aluminum samples, corrections

## Abstract

Nonlinear ultrasound is often employed to assess microdamage or nonlinear elastic properties of a material, and the nonlinear parameter is commonly used to quantify damage sate and material properties. Among the various factors that influence the measurement of nonlinear parameters, maintaining a constant contact pressure between the receiver and specimen is important for repeatability of the measurement. The use of an air-coupled transducer may be considered to replace the contact receiver. In this paper, a method of measuring the relative and absolute nonlinear parameters of materials is described using an air-coupled transducer as a receiver. The diffraction and attenuation corrections are newly derived from an acoustic model for a two-layer medium and the nonlinear parameter formula with all corrections is defined. Then, we show that the ratio of the relative nonlinear parameter of the target sample to the reference sample is equal to that of the absolute nonlinear parameter, and this equivalence is confirmed by measurements on three systems of aluminum samples. The proposed method allows the absolute measurement of the nonlinear parameter ratio or the nonlinear parameter without calibration of the air-coupled receiver and removes restrictions on the selection of reference samples.

## 1. Introduction

The acoustic nonlinear parameter becomes a powerful tool in the nondestructive evaluation field as a measure of material nonlinearity and damage state in structural components. This parameter can quantitatively be obtained by harmonic generation measurements. The most widely used technique is the finite amplitude method, in which a high power wave of a monochromatic frequency propagates through a nonlinear medium introduces distortions, resulting in the generation of higher harmonics. Harmonic generation measurements for evaluating nonlinear parameters can be conducted using several wave types, different generation and detection methods, and a variety of experimental set-ups. The general experimental procedure is similar in all cases, where an ultrasonic tone burst at frequency *ω* is launched from the emitting transducer, it propagates some distance through the material, and the response is measured by the receiving transducer—specifically, the amplitudes of the fundamental and second harmonic waves are extracted from the frequency response of the received signal.

Contact piezoelectric transducers are most commonly used as emitting transducers of longitudinal waves in the through-transmission setup. Various types of detectors can be used as receiving transducers of both fundamental and second harmonic wave components. Detection of second harmonic generation measurements using longitudinal waves has been conducted with contact piezoelectric transducers [1,2,3,4,5,6], capacitive transducers [7,8,9,10,11,12,13,14], and laser interferometers [15,16,17,18]. An absolute measurement of material nonlinearity is possible using either capacitive transducers [9,12] or contact piezoelectric transducers using a calibration procedure [19,20] in which the absolute displacement amplitude of the fundamental and second harmonic waves can be measured.

The various methods used to detect nonlinear signals suffer from significant limitations. Piezoelectric contact transducers, while being easy to use in many ways, are heavily influenced by contact conditions between the transducer and sample surface, so that application of a consistent force is crucial to measurement repeatability.

Noncontact detection methods such as capacitive receivers and laser interferometers are more desirable from the practical point of view, but they also have some drawbacks. Laser interferometry requires a mirror-finished sample surface and relies on complicated optics to maximize sensitivity. Careful preparation of sample surfaces is also very important in the capacitive receiver technique, requiring an optically flat and parallel sample surface over the entire receiver area and a small gap spacing of only a few microns [21,22].

Compared to existing noncontact detection methods such as capacitive receivers or laser interferometers, air-coupled transducers are easy to handle, significantly less expensive, and robust relative to surface conditions. As far as we know, only relative measurements were possible with air-coupled transducers, mainly because they are difficult to calibrate for use in nonlinear measurements. Existing calibration techniques such as self-reciprocity methods are not directly applicable to the air-coupled receivers because of high ultrasonic attenuation loss in air. Consequently, most second harmonic generation measurements were limited to relative measurements. Recently, air-coupled transducers have been applied to second harmonic generation measurements as an efficient detection tool for Rayleigh and Lamb waves [23]. The air-coupled transducer detects a longitudinal wave in air that is leaked from the propagating Rayleigh wave or Lamb wave in the sample. Torello et al. [24] reported a hybrid acoustic modeling and experimental approach to air-coupled transducer calibration and the use of this calibration in a model-based optimization to determine the absolute nonlinear parameter of representative materials. More recently, Li et al. [25] proposed a comparative approach where four separate experimental setups are used to obtain the sensitivity or the transfer function of an air-coupled ultrasonic receiver and to measure material nonlinear parameter.

The purpose of this paper is to develop a new technique for absolute measurement of material nonlinearity using air-coupled receivers without separate receiver calibration. First, an acoustic model for a two-layer medium composed of solid specimen and air is considered, and the diffraction and attenuation corrections are derived from the wave field analysis. These corrections convert the measured fundamental and second harmonic amplitudes to the plane wave amplitudes in nonlinear parameter calculations. Next, it is shown that the ratio of the relative nonlinear parameter of the target specimen to the reference specimen ($\beta^{\prime }/{\beta^{\prime }}_{\mathrm{ref}}$) is the same as the absolute nonlinear parameter ratio of the two specimens ($\beta /\beta_{\mathrm{ref}}$). This equivalence allows an absolute comparison of the material nonlinearity between different materials from the measurement of relative nonlinear parameter. Furthermore, the absolute nonlinear parameter of a target specimen (β) can be obtained by measuring the relative nonlinear parameter ratio, $\beta^{\prime }/{\beta^{\prime }}_{\mathrm{ref}}$, if the absolute nonlinear parameter of the reference specimen ($\beta_{\mathrm{ref}}$) is available. A nonlinear ultrasonic testing system including an air-coupled receiver is constructed, and the relative and absolute nonlinear parameters are measured for aluminum specimens of three different types: Al2024, Al6061, and Al7075. The equivalence between the relative and absolute parameter ratios is verified through experimental results.

Section 2 describes the nonlinear acoustic model for a two-layer medium composed of solid and air and defines the nonlinear parameter formula with necessary corrections. The equivalence of relative nonlinear parameter ratio and absolute nonlinear parameter ratio is also demonstrated. Simulation results on the received displacement and diffraction and attenuation corrections are provided in Section 3. Section 4 introduces the experimental setup and specimens, and Section 5 presents the experimental results. Conclusions are drawn in Section 6.

## 2. Sound Beam Fields and Nonlinear Parameter

In order to calculate the received wave fileds and to define the nonlinear parameter, we need an appropriate model equation for finite amplitude radiation that takes into account the combined effects of nonlinearity, diffraction, and attenuation. For this purpose, a Westervelt-type equation similar to the Westervelt equation for sound beams of fnite amplitude in fuids can be used for longitudinal wave motion in isotropic solids [26,27]. Such equation can be obtained from the Westervelt equation in a manner similar to the derivation of the KZK-type equation from the KZK equation [28].

Applying the quasilinear theory to the Westervelt-type equation yields the governing equations for the fundamental and second harmonic waves for axisymmetric sound source. The Green’s function approach is a convenient method for constructing the integral solutions to these equations. Then, the solutions can be obtained by integrating over the product of the Green’s function and the appropriate source function to sum up the contributions from all source points. To calculate the received ultrasonic fields by a finite radius receiver, the concept of field averaging can be used.

In general, the accurate determination of the nonlinear parameter in a wide range of experimental conditions requires attenuation and diffraction corrections to the plane wave solution. The attenuation correction for the fundamental wave is well known [29]. The attenuation correction for the second harmonic wave generated by the focring of the propagating fundamental wave is also well known [30]. For wave propagation in a multi-layered medium, attenuation correction can be defined for each layer, and the total attenuation correction for the entire medium can be obtained by simply multiplying the correction in each layer.

A closed form of the diffraction correction for the fundamental wave was found by Rogers and Van Buren [31] when the transmitter and receiver are of the same size. However, the integral solutions can also be used to numerically calculate the diffraction correction of the fundamental wave for a more general transmitter-receiver combination [32]. The diffraction correction is defined as the magnitude of the fundamental or second harmonic wave divided by the corresponding plane wave solution. Similarly, the diffraction correction for the second harmonic wave can be found numerically from the magnitude of the second harmonic wave and the plane wave solution of the second harmonic wave [32]. The concept of diffraction correction for a single medium can be extended to a two-layer medium that is covered in this study.

In this work, the diffraction corrections for the fundamental and second harmonic waves are developed using the integral solutions of the Westervelt-type equation [26,27]. The diffraction correction for a single medium is extended to a two-layer medium consisting of a solid layer (specimen) and an air layer. The analytical diffraction correction can be efficiently used for a wide range of two-layer media and transmitter-receiver geometries.

**Figure 1:**
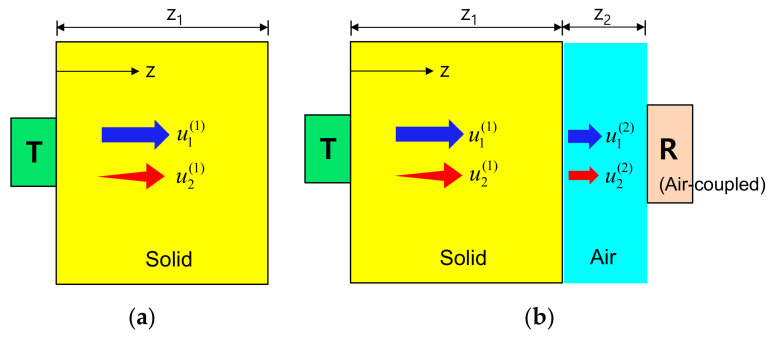
(**a**) Fundamental wave propagation and second harmonic generation in a solid specimen, and (**b**) Transmission at the solid-air interface, propagation in the air, and reception by an air-coupled ultrasonic receiver.

First, using the approach mentioned above, we construct the displacement field expressions for the fundamental and second harmonic waves propagating in the solid specimen depicted in Figure 1a. The propagation in a single medium is then extended to the analysis of propagation in a two-layer medium composed of the solid specimen and air, and the displacement fields received by an air-coupled transducer are obtained, as shown in Figure 1b. Finally, we will define the diffraction corrections for the fundamental and second harmonic waves in the two-layer medium, and derive a nonlinear parameter expression modified by the attenuation and diffraction corrections.

### 2.1. Sound Beam Solutions for a Single Medium

Referring to Figure 1a, the fundamental displacement field in medium 1, $u_{1}^{\left(1\right)}$, in the forward propagation ($0\leq z\leq z_{1}$) region and the generated second harmonic displacement field, $u_{2}^{\left(1\right)}$, due to the finite amplitude radiation at the transmitter can be expressed as: [26]

(1) $$u_{1}^{\left(1\right)}(x,y,z)=-2ik^{\left(1\right)}\int_{-\infty }^{+\infty }\int_{-\infty }^{+\infty }u_{1}(x^{\prime },y^{\prime },0)\;G_{1}^{\left(1\right)}(x,y,z|x^{\prime },y^{\prime },0)\;dx^{\prime }dy^{\prime }\;\;\;$$

(2) $$u_{2}^{\left(1\right)}(x,y,z)=\frac{\beta k^{\left(1\right)}}{2{\left(c^{\left(1\right)}\right)}^{2}}\int_{0}^{z}\int_{-\infty }^{+\infty }\int_{-\infty }^{+\infty }{\left[u_{1}^{\left(1\right)}(x^{\prime },y^{\prime },z^{\prime })\right]}^{2}\;G_{2}^{\left(1\right)}\left(x,y,z|x^{\prime },y^{\prime },z^{\prime }\right)\;dx^{\prime }dy^{\prime }dz^{\prime }\;\;\;$$

Here, β is the nonlinear parameter of the solid specimen, and defined as $\beta =-\left(3+\frac{C_{111}}{C_{11}}\right)$ where $C_{11}$ and $C_{111}$ are the second and third order elastic constants, respectively. In Equations (1) and (2), $k_{}^{\left(1\right)}$ and $c^{\left(1\right)}$ are the wave number and wave velocity of medium 1, respetively. The Green’s function can be obtained as

(3) $$G_{n}^{\left(1\right)}(x,y,z|x^{\prime },y^{\prime },z^{\prime })=\frac{1}{4\pi r_{n}}\exp(ink^{\left(1\right)}r_{n})\;\;,n=1,\;2$$

where $r_{1}=\sqrt{{\left(x-x^{\prime }\right)}^{2}+{\left(y-y^{\prime }\right)}^{2}+z^{2}}$ and $r_{2}=\sqrt{{\left(x-x^{\prime }\right)}^{2}+{\left(y-y^{\prime }\right)}^{2}+{\left(z-z^{\prime }\right)}^{2}}$ is the distance from the sound source point $\left(x^{\prime },y^{\prime },0\right)$ to the target point (x,y,z). The attenuation effect is not included in the Green function and will be treated separately. A constant displacement $U_{0}$ is prescribed over the surface $S^{\prime }$ of a circular piston transducer of radius a:

(4) $$\left\{\begin{array}{l} u_{1}\left(x^{\prime },y^{\prime },z^{\prime }=0\right)=U_{0},\;\;\;\;\;\;\;\;{x^{\prime }}^{2}+{y^{\prime }}^{2}\leq a^{2}\\ u_{2}\left(x^{\prime },y^{\prime },z^{\prime }=0\right)=0 \end{array}\right.\;.$$

Note that the range of wave propagation distance in Equations (1) and (2) is $0\leq z\leq z_{1}$. Equation (1) provides an exact solution to calculate the fundamental wave field, and represents a superposition of spherical waves radiating from the point sources distributed on the source plane $z^{\prime }=0$. When the second harmonic wave field is calculated using Equation (2), the Green’s function used in this integral includes contribution of the element $dV^{\prime }=dx^{\prime }dy^{\prime }dz^{\prime }$ of the virtual source formed by the fundamental wave field. If a displacement source such as Equation (4) is defined, these equations can be used to obtain the fundamental and second harmonic displacement fields.

Equations (1) and (2) provide the exact displacement solutions for the fundamental and second harmonic waves for a planar, circular P-wave transducer radiating at normal incidence in medium 1, and can be expressed in the following form:

(5) $$u_{1}^{\left(1\right)}(x,y,z)=\left[U_{1}^{plane}\right]\left[D_{1}^{\left(1\right)}(a,x,y,z)\right]$$

(6) $$u_{1}^{\left(2\right)}(x,y,z)=\left[U_{2}^{plane}\right]\left[D_{2}^{\left(1\right)}(a,x,y,z)\right]$$

where the first square bracket on the right-hand side represents the pure plane wave solutions, and the second bracket the diffraction corrections. The plane wave solutions are given by:

(7) $$U_{1}^{plane}=U_{0}\exp(ik^{\left(1\right)}z)$$

(8) $$U_{2}^{plane}=\frac{{\left(k^{\left(1\right)}\right)}^{2}\beta zU_{0}^{2}}{8}\exp(2ik^{\left(1\right)}z)$$

and the diffraction corrections are defined as $D_{1}^{\left(1\right)}=u_{1}^{\left(1\right)}(x,y,z)/U_{1}^{plane}$ and $D_{2}^{\left(1\right)}=u_{2}^{\left(1\right)}(x,y,z)/U_{2}^{plane}$.

### 2.2. Sound Beam Solutions for a Two-Layer Medium

Prior to further acoustic modeling and simulation, it is necessary to understand the transmission, generation, propagation, and reception of the fundamental and second harmonic waves in a two-layer medium consisting of solid and air. The fundamental wave and the second harmonic wave generated by the propagating fundamental wave both propagate in the solid medium (Figure 1a) and these two waves are transmitted at the solid-air interface and then propagate into the air (Figure 1b). Due to the small intensity of the transmitted fundamental wave resulting from the very low transmission coefficient and the very high attenuation loss in the air, it is difficult to meet the conditions for generating a new second harmonic wave in the air. Therefore, the generation of the second harmonic that may be newly generated by the transmitted fundamental wave in the air is ignored. Similarly, it is assumed that the second harmonic wave in the solid propagates only as the second harmonic in the air without generating other waves. These are schematically shown in Figure 1b.

Approximate methods such as the multi-Gaussian beam (MGB) model may be used to formulate the wave fields in the two-layer medium. The MGB model is based on the paraxial approximation and is known to be very computationally efficient [29]. This model accurately calculates the sound beam fields of an ultrasonic transducer at distances of approximately one transducer diameter or greater from the transducer face. In the two-layer medium covered here, however, the distance from the solid-air interface to the air-coupled receiver is only a few millimeters, which is very short. Therefore, in this case, the paraxial MGB solutions may fail in providing accurate beam field results, especially the diffraction corrections. Therefore, exact solutions are required for modeling a planar, circular P-wave transducer radiating at normal incidence in the solid-air interface. The integral solutions of Equations (1) and (2) are extended to the wave field analysis in the second medium.

Based on this observation, the propagating fundamental and second harmonic waves in the second medium (air) can be found by using the results of Equations (1) and (2) at $z=z_{1}$ as new sound sources for radiation into the second medium. Then, the integral solutions become:

(9) $$u_{1}^{\left(2\right)}(x,y,z)=T_{12}\times \left[-2ik^{\left(2\right)}\int_{-\infty }^{+\infty }\int_{-\infty }^{+\infty }u_{1}^{\left(1\right)}(x^{\prime },y^{\prime },z_{1})\;G_{1}^{\left(2\right)}(x,y,z|x^{\prime },y^{\prime },z_{1})\;dx^{\prime }dy^{\prime }\;\;\right]\;$$

(10) $$u_{2}^{\left(2\right)}(x,y,z)=T_{12}\times \left[-2ik^{\left(2\right)}\int_{-\infty }^{\infty }\int_{-\infty }^{\infty }u_{2}^{\left(1\right)}(x^{\prime },y^{\prime },z_{1})G_{2}^{\left(2\right)}(x,y,z|x^{\prime },y^{\prime },z_{1})dx^{\prime }dy^{\prime }\right].$$

In the above equation, $T_{12}$ is the transmission coefficient given by $T_{12}=\frac{Z_{2}}{Z_{1}+Z_{2}}$ where $Z_{1}$ and $Z_{2}$ are the acoustic impedeances of the medium 1 and 2, respectively. The linear transmission is assumed for the nonlinear second harmonic wave in Equation (10), but this is a good approximation for the solid-air interface. Note that the range of wave propagation distance in these equations is $z_{1}\leq z\leq z_{2}$. These equations can also be written in the quasi-plane wave form similar to Equations (5) and (6). The diffraction effects of the fundamental and second harmonic waves propagating in the second medium are defined as $D_{1}^{\left(2\right)}=u_{1}^{\left(2\right)}(x,y,z)/U_{1}^{plane},\;D_{2}^{\left(2\right)}=u_{2}^{\left(2\right)}(x,y,z)/U_{2}^{plane}$, where the plane wave solutions are given by $U_{1}^{plane}=U_{0}T_{12}\exp(ik^{\left(1\right)}z_{1}^{}+ik^{\left(2\right)}z)$, $U_{2}^{plane}=\frac{{\left(k_{}^{\left(1\right)}\right)}^{2}\beta z_{1}U_{0}^{2}}{8}T_{12}\exp(i2k^{\left(1\right)}z_{1}+i2k^{\left(2\right)}z)$. The detailed expressions for the plane wave solutions and diffraction corrections are given later together with the attenuation corrections.

Finally, to calculate the received displacement at distance $z_{2}$ by a circular air-coupled transducer of radius b, the concept of average field can be used and calculated as follows:

(11) $${\tilde{u}}_{n}^{\left(m\right)}(z_{2})=\frac{1}{\pi b^{2}}\int_{S}^{}u_{n}^{\left(m\right)}(x,y,z_{2})\;dS,n=1,2$$

where $u_{n}^{\left(m\right)}(x,y,z_{2})$ is computed from Equations (9) and (10). Substituting Equations (9) and (10) into Equation (11) and performing some manipulation, the received displacement fields in the second medium can be written in the following form:

(12) $${\tilde{u}}_{1}(z_{2})=\left[U_{0}T_{12}\exp(ik^{\left(1\right)}z_{1}^{}+ik^{\left(2\right)}z_{2}^{})\right]\left[D_{1}^{}(a,b,z_{1},z_{2})\right]$$

(13) $${\tilde{u}}_{2}(z_{2})=\left[\frac{{\left(k^{\left(1\right)}\right)}_{}^{2}\beta z_{1}U_{0}^{2}}{8}T_{12}\exp(i2k^{\left(1\right)}z_{1}+i2k^{\left(2\right)}z_{2})\right]\left[D_{2}(a,b,z_{1},z_{2})\right]$$

where the first term in the bracket in each equation represents the plane wave solution and $D_{n}$ denotes the diffraction correction for the fundamental (n=1) and second harmonic (n=2) waves. The detailed expressions of $U_{n}^{plane}$ and $D_{n}$ in Equations (12) and (13) are given by

(14) $$U_{1}^{plane}=U_{0}T_{12}\exp(ik^{\left(1\right)}z_{1}^{}+ik^{\left(2\right)}z_{2}^{})$$

(15) $$U_{2}^{plane}=\frac{{\left(k^{\left(1\right)}\right)}_{}^{2}\beta z_{1}U_{0}^{2}}{8}T_{12}\exp(i2k^{\left(1\right)}z_{1}+i2k^{\left(2\right)}z_{2})$$

(16) $$D_{1}^{}(a,b,z_{1},z_{2})=\frac{{\tilde{u}}_{1}(z_{2})}{U_{1}^{plane}}$$

(17) $$D_{2}(a,b,z_{1},z_{2})=\frac{{\tilde{u}}_{2}(z_{2})}{U_{2}^{plane}}.$$

The diffraction corrections given by Equations (16) and (17) are defined as the received average displacement of the fundamental or second harmonic wave propagated through the entire medium divided by the amplitude of the plane wave involved in the same propagation process. In the current solid-air medium, the fundamental wave is generated from the start of radiation and continues to propagate only as the fundamental wave in medium 1 (solid) and 2 (air). However, the second harmonic wave is generated and propagated by the propagating fundamental wave in the first medium, and then propagates as a fundamental wave of frequency *2f* in the second medium.

The attenuation correction for the fundamental and second harmonic waves is well known in the second harmonic generation process in a single medium and can be considered separately since it only affects the amplitude of the propagating wave. Thus, the attenuation correction that occurs over the entire propagation process can be obtained by successively multiplying the attenuation correction of each layer. With the inclusion of the attenuation corrections, the final expressions of the received displacement fields in the second medium are obtained as:

(18) $${\tilde{u}}_{1}(z_{2})=\left[U_{0}T_{12}\exp(ik^{\left(1\right)}z_{1}^{}+ik^{\left(2\right)}z_{2}^{})\right]\left[D_{1}^{}(a,b,z_{1},z_{2})\right]\left\{\left[M_{1}^{\left(1\right)}(\alpha_{1}^{\left(1\right)},z_{1})\right]\left[M_{1}^{\left(2\right)}(\alpha_{1}^{\left(2\right)},z_{2})\right]\right\}$$

(19) $${\tilde{u}}_{2}(z_{2})=\left[\frac{{\left(k^{\left(1\right)}\right)}_{}^{2}\beta z_{1}U_{0}^{2}}{8}T_{12}\exp(i2k^{\left(1\right)}z_{1}+i2k^{\left(2\right)}z_{2})\right]\left[D_{2}(a,b,z_{1},z_{2})\right]\left\{\left[M_{2}^{\left(1\right)}(\alpha_{1}^{\left(1\right)},\alpha_{2}^{\left(1\right)},z_{1})\right]\left[M_{2}^{\left(2\right)}(\alpha_{2}^{\left(2\right)},z_{2})\right]\right\}$$

where $\alpha_{1}^{\left(m\right)}$ and $\alpha_{2}^{\left(m\right)}$ are the attenuation coefficients at the fundamental and second harmonic frequencies in the *m*th medium, respectively. The detailed expressions of $M_{n},\;n=1,2$ appearing in the above equations are given by:

(20) $$\left[M_{1}^{\left(1\right)}\right]\left[M_{1}^{\left(2\right)}\right]=M_{1}=\left[\exp(-\alpha_{1}^{\left(1\right)}z_{1})\right]\left[\exp(-\alpha_{1}^{\left(2\right)}z_{2})\right]$$

(21) $$\left[M_{2}^{\left(1\right)}\right]\left[M_{2}^{\left(2\right)}\right]=M_{2}=\left[\frac{\exp(-2\times \alpha_{1}^{\left(1\right)}z_{1})-\exp(-\alpha_{2}^{\left(1\right)}z_{1})}{(\alpha_{2}^{\left(1\right)}-2\alpha_{1}^{\left(1\right)})z_{1}}\right]\exp\left[-\alpha_{2}^{\left(2\right)}z_{2}\right].$$

The attenuation correction of the fundamental wave in each layer is found to be the same as the simple exponential attenuation law in linear acoustics. The attenuation correction of the second harmonic wave in the first solid layer shows a somewhat complicated behavior, since it will grow with propagation distance due to the nonlinear interaction effects, and will also decrease due to attenuation effects.

### 2.3. Definition of Nonlinear Parameter

The nonlinear parameter, β, can be found from Equations (18) and (19) by cancelling $U_{0}$ in both terms:

(22) $$\beta =\left[\frac{8U_{2}\left(z_{2}\right)}{{\left(k^{\left(1\right)}\right)}_{}^{2}z_{1}U_{1}^{2}\left(z_{2}\right)}\right]\;\;\;\left[\frac{M_{1}^{2}\left(\alpha_{1}^{\left(1\right)},\alpha_{1}^{\left(2\right)}z_{1},z_{2}\right)}{M_{2}\left(\alpha_{1}^{\left(1\right)},\alpha_{2}^{\left(1\right)},\alpha_{2}^{\left(2\right)},z_{1},z_{2}\right)}\right]\left[\frac{{\left|D_{1}\left(a,b,z_{1},z_{2}\right)\right|}^{2}}{\left|D_{2}\left(a,b,z_{1},z_{2}\right)\right|}\right]\;$$

where $U_{1}$ and $U_{2}$ are the displacement amplitudes of the received fundamental and second harmonic waves, respectively. The first square bracket in Equation (22) represents the uncorrected nonlinear paramter, and the second and third brackets represent the attenuation correction and diffraction correction, respectively. The nonlinear parameter β is defined using the amplitude of the fundamental and second harmonic waves of the pure plane wave. However, the amplitude of the measured wave in the real environment deviates from the pure plane wave due to material attenuation and finite size transducers. Therefore, attenuation and diffraction corrections are required to convert the actually measured wave amplitude into the plane wave amplitude.

The absolute measurement of the received displacement generally requires the use of a calibrated receiving transducer. In case of an air-coupled receiver, however, the reciprocity-based calibration [19,20] is very difficult to perform because of high attenuation in the air. Due to the difficulty of obtaining calibration measurements, it is possible to use an electrical output signal instead of the received displacement in the measurement of nonlinear parameters. If the amplitudes of received fundamental and second harmonic waves are denoted by the electrical current output signals denoted by $A_{1}(\omega )$ and $A_{2}(\omega )$, respectively, then the relative nonlinear parameter, $\beta^{\prime }$, with the inclusion of attenuation and diffraction corrections can be expressed as:

(23) $$\beta^{\prime }=\left[\frac{8A_{2}}{{\left(k_{}^{\left(1\right)}\right)}^{2}z_{1}A_{1}^{2}}\right]\;\;\;\left[\frac{M_{1}^{2}}{M_{2}}\right]\left[\frac{{\left|D_{1}\right|}^{2}}{\left|D_{2}\right|}\right]\;.$$

Now we define the relative nonlinear parameter ratio of a target sample to a reference sample as follows:

(24) $$\frac{\beta^{\prime }}{{\beta^{\prime }}_{\mathrm{ref}}}=\frac{\left[\frac{8A_{2}}{{\left(k_{}^{\left(1\right)}\right)}^{2}z_{1}A_{1}^{2}}\right]\;\;\;\left[\frac{M_{1}^{2}}{M_{2}}\right]\left[\frac{{\left|D_{1}\right|}^{2}}{\left|D_{2}\right|}\right]}{{\left(\left[\frac{8A_{2}}{{\left(k_{}^{\left(1\right)}\right)}^{2}z_{1}A_{1}^{2}}\right]\;\;\;\left[\frac{M_{1}^{2}}{M_{2}}\right]\left[\frac{{\left|D_{1}\right|}^{2}}{\left|D_{2}\right|}\right]\right)}_{\mathrm{ref}}}\;.$$

By including attenuation and diffraction corrections in the denominator of Equation (24), the restrictions on the type and thickness of the reference specimen can be removed and any specimen can be used. In Equation (24), the amplitude of the electrical current signal can be expressed in terms of the absolute displacement amplitude by using the relationship between these two quantities, A(ω)=U(ω)/H(ω), where H(ω) is the transfer function of the receive transducer [33]. Then, it can be easily shown from Equation (25) below that the relative nonlinear parameter ratio is equal to the absolute nonlinear parameter ratio, since the ratio of the transfer function $H_{1}^{2}/H_{2}$ is a characteristic of the receive transducer and does not depend on the type of specimen.

(25) $$\frac{\beta^{\prime }}{{\beta^{\prime }}_{\mathrm{ref}}}=\frac{\left[\frac{8U_{2}}{{\left(k_{}^{\left(1\right)}\right)}^{2}z_{1}U_{1}^{2}}\right]\;\;\left[\frac{H_{1}^{2}}{H_{2}}\right]\;\left[\frac{M_{1}^{2}}{M_{2}}\right]\left[\frac{{\left|D_{1}\right|}^{2}}{\left|D_{2}\right|}\right]}{{\left(\left[\frac{8U_{2}}{{\left(k_{}^{\left(1\right)}\right)}^{2}z_{1}U_{1}^{2}}\right]\;\;\;\left[\frac{H_{1}^{2}}{H_{2}}\right]\left[\frac{M_{1}^{2}}{M_{2}}\right]\left[\frac{{\left|D_{1}\right|}^{2}}{\left|D_{2}\right|}\right]\right)}_{\mathrm{ref}}}=\frac{\beta }{\beta_{\mathrm{ref}}}$$

Therefore, the ratio of the absolute nonlinear parameters between two different materials can be obtained by measuring the ratio of the relative nonlinear parameters, which then allows a quantitative comparison of the material nonlinearity between different materials. Equation (25) also demonstrates that the absolute nonlinear parameter of a target specimen (β) can be obtained by measuring the relative nonlinear parameter ratio of a target sample to a reference sample ($\beta^{\prime }$/${\beta^{\prime }}_{\mathrm{ref}}$) if the absolute nonlinear parameter of the reference specimen ($\beta_{\mathrm{ref}}$) is available. This observation can be written as

(26) $$\beta =\left(\frac{\beta^{\prime }}{{\beta^{\prime }}_{\mathrm{ref}}}\right)\beta_{\mathrm{ref}}.$$

## 3. Simulation Results

In the measurement of the relative or absolute nonlinear parameter of a solid specimen using an air-coupled transducer, the diffraction and attenuation corrections are important factors affecting the measurement results. In particular, the diffraction correction equations for a two-layer medium of solid-air is complicated and computationally heavy due to multiple integrations involved. Therefore, the calculation of accurate diffraction correction is the main purpose of the wave field simulation.

The acoustic parameters of the two-layered medium used in the calculation of wave fileds are listed in Table 1. The acoustic properties of Al6061 including the nonlinear parameter β were taken from the measurement [33]. For air, the wave speed and density are the values at temperature 20 °C and the value of *β* was taken from [34]. The frequency-dependent attenuation coefficient of air was taken from [35]. In addition, the source displacement used is 1.0 m, and the fundamental frequency used is 2 MHz. The diameters of transmit and receive transducers are all 12.7 mm. The propagation distance or the layer thickness of layers 1 and 2 is 12 cm and 0.5 cm, respectively.

In the wave field calculation, the received displacement, diffraction correction, and attenuation correction for the fundamental wave and second harmonic waves were obtained over the entire propagation process of the two-layer medium. In the calculation of the received displacement, both the transmission coefficient and the attenuation effect were neglected.

**Figure 2:**
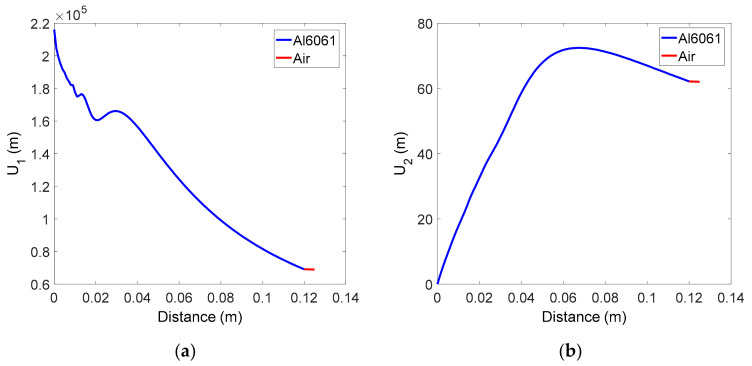
Variation of received average displacement in the two-layer medium of solid-air: (**a**) Fundamental wave, and (**b**) Second harmonic wave.

Figure 2a,b respectively shows the received displacement amplitude of the fundamental and second harmonic waves, calculated using Equations (9) and (10). The behavior of the received displacement in the solid specimen is not unfamiliar and is similar to the results observed in previous studies [36]. The distance between the solid-air interface and the receiver is 0.5 cm, which is very short, the transmission coefficient was assumed to be 1, and the attenuation in the air layer was neglected, so the displacement received in the air layer remains almost the same and is equal to the displacement at the end of the solid specimen. Thus, the diffraction correction at the receiver position can be replaced by the value at the end of the solid specimen.

**Figure 3:**
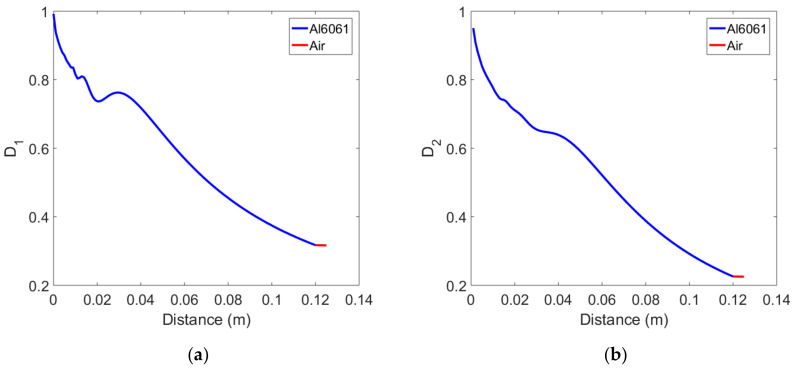
Variation of diffraction correction in the two-layer medium composed of solid and air: (**a**) Fundamental wave, and (**b**) Second harmonic wave.

Diffraction corrections are defined by Equations (16) and (17) as the division of the received average displacement at each propagation distance by the displacement of the plane wave at the same distance without considering the effects of attenuation and transmission interface. Diffraction corrections were calculated for the Al6061, 12 cm thick sample and the results are shown in Figure 3 as a function of the propagation distance. Figure 3a is the plot of ${\tilde{D}}_{1}$ of the fundamental wave, and Figure 3b is the plot of ${\tilde{D}}_{2}$ of the second harmonic wave. Since the amplitude of the plane fundamental wave is a constant at all propagation distances, the overall behavior of ${\tilde{D}}_{1}$ looks the same as that of the received fundamental wave shown in Figure 2a. Since the amplitude of the plane second harmonic wave increases linearly with the propagation distance, ${\tilde{D}}_{2}$ decreases with the propagation distance in the solid layer and then looks like Figure 3b. ${\tilde{D}}_{2}$ is smaller than ${\tilde{D}}_{1}$ at the same propagation distance. As pointed out earlier, the diffraction correction in the air layer can be replaced by the value at the end of the solid layer, in which case the two-layer medium composed of solid and air can be treated as a single solid medium.

**Figure 4:**
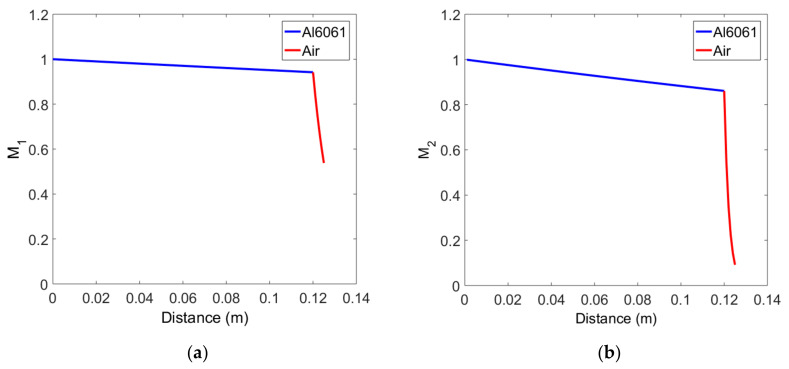
Variation of attenuation correction in the two-layer medium of solid-air: (**a**) Fundamental wave, and (**b**) Second harmonic wave.

Attenuation corrections in each layer of the solid-air medium are defined by Equations (20) and (21). Using these equations, attenuation corrections were calculated for the Al6061, 12 cm thick sample, and the results for the fundamental and second harmonics as a function of propagation distance are shown in Figure 4a,b, respectively. In the first solid layer, the fundamental wave exponentially decreases, and the second harmonic shows a slightly greater decrease in amplitude at the same propagation distance than the fundamental wave. The attenuation correction in the second air layer shows a very rapid decrease compared to the first solid layer in both the fundamental wave and the second harmonic wave, which is due to the very high attenuation coefficient of the air layer. The amplitude reduction of the second harmonic due to the attenuation is very severe compared to the fundamental wave, which is caused by the attenuation coefficient proportional to the square of the frequency.

## 4. Measurement of Relative Nonlinear Parameter $\beta^{\prime }$

### 4.1. Experiment

**Figure 5:**
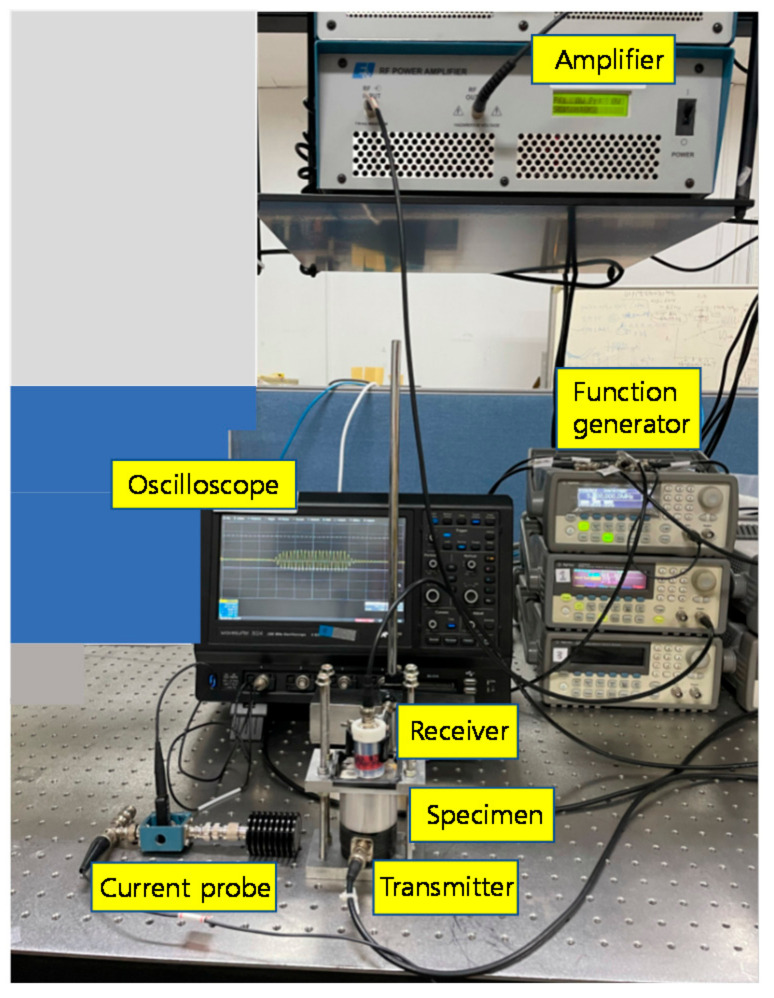
Experimental setup for harmonic generation measurement in the through-transmission mode using a contact transmitter and an air-coupled receiver.

Figure 5 shows the experimental setup for second harmonic generation measurement in the through-transmission mode using an air-coupled transducer as a receiver. The transmit transducer is a single crystal LiNbO_3_ (Boston Piezo-Optics, Bellingham, MA, USA) of 12.7 mm diameter and 2 MHz center frequency. The receive transducer is an air-coupled transducer (NCT2-D13, Ultran Group, State College, PA, USA) of 12.7 mm diameter and 2 MHz center frequency. The two transducers are aligned with each other for maximum output signal capture. The entire propagation region is composed of two layers: solid specimen and air. The air gap from the solid specimen end to the receiver face is fixed at 5 mm. In the transmission side, a toneburst of 20 cycles tuned to the fundamental frequency (2 MHz) is supplied by a function generator (33250A, Agilent Technologies, Inc., Santa Clara, CA, USA), and then amplified by a linear amplifier (2100L, Electronics & Innovation, Ltd., Rochester, NY, USA) to provide a high-power monochromatic toneburst for harmonic generation in the solid specimen. In the reception side, the receive transducer is directly connected with a current probe (Tektronix CT-2, Tektronix, Inc., Wilsonville, OR, USA) and digitized using a digital oscilloscope (LT 332, LeCroy, Inc., Chestnut Ridge, NY, USA).

The purpose of this experiment is to accurately obtain the relative nonlinear parameter, $\beta^{\prime }$, of aluminum specimens by applying the attenuation and diffraction corrections to the measured current output. Then, the ratio of relative nonlinear parameters of two different materials can be obtained accordingly, which is equal to the ratio of absolute nonlinear parameters.

### 4.2. Specimen

Three types of aluminum specimens: Al2024, Al6061 and Al7075, were selected, and five different thicknesses in each Al type were prepared: 4 cm, 6 cm, 8 cm, 10 cm, and 12 cm. The shape of the specimen is a block of circular cross section, 5 cm in diameter.

**Figure 6:**
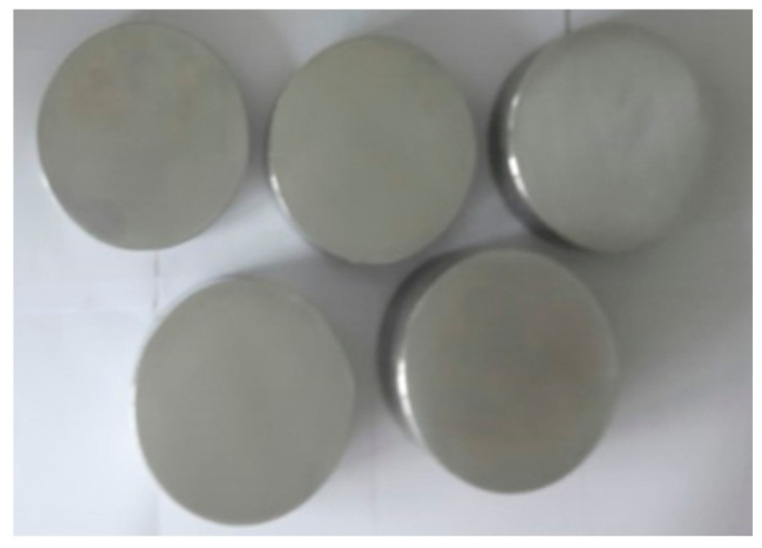
Aluminum specimens after surface machining.

It has been known that the measured value of nonlinear parameter is affected by the parallelism of the top and bottom surfaces of the specimen. The surface roughness of the specimen also has a direct influence on the accuracy of the measurement of nonlinear parameters [37]. In order to minimize the effect of surface roughness, it is necessary to maintain the same surface roughness on each specimen as much as possible. The prepared specimens were machined so that the upper and lower surfaces were parallel. The surface roughness of each specimen was maintained at the same level as possible using a metal abrasive. Table 2 shows the surface roughness of each specimen. The surface roughness was measured 5 times per specimen and then the mean value was calculated. The mean roughness for all specimens is 0.11. Figure 6 shows the representative aluminum specimens after surafce machining.

## 5. Results and Discussion

**Figure 7:**
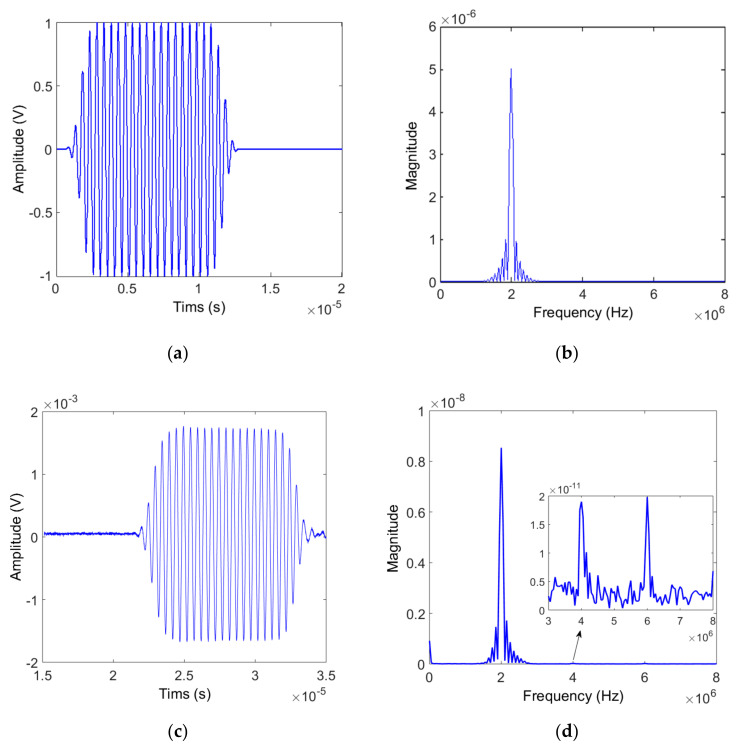
Input signal (**a**) Waveform and (**b**) Spectrum, and output signal (**c**) Waveform and (**d**) Spectrum.

Figure 7a,b show the input waveform and its frequency spectrum. Here, the toneburst waveform consisting of twenty cycles produces a narrowband spectrum with a center frequency of 2 MHz. In nonlinear ultrasonic measurements, the second harmonic amplitude is generally two or three orders of magnitude lower than the fundamental wave amplitude. When using the air-coupled receiver, it may be more difficult to receive the second harmonic component because of low transmission efficiency at the solid-air interface and high attenuation loss in the air. Therefore, the possibility of receiving the second harmonic component was confirmed through spectrum analysis of the output signal received from each specimen. Figure 7c,d show the received signal and its frequency spectrum measured on Al6061, 12 cm thick specimen. From the frequency spectrum, we can see that not only the second harmonic component but also the third harmonic component are well received.

**Figure 8:**
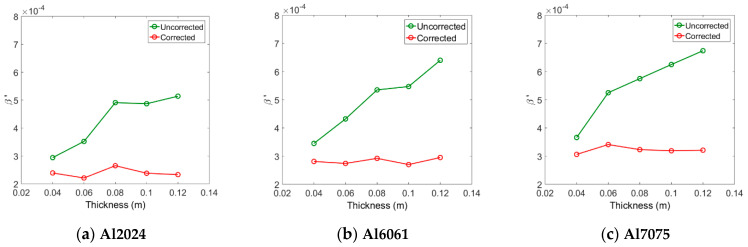
Measured $\beta^{\prime }$ before and after all corrections for three different aluminum systems.

Next, the relative nonlinear parameter $\beta^{\prime }$ was measured for each specimen. During the experiment, it was observed that the output signal was very noisy due to the influence of external variables such as vibration of the experiment table and alignment of the probes. Therefore, in order to increase the signal-to-noise ratio of the output signal, 500 summed averages were used. Figure 8 shows the relative nonlinear parameter $\beta^{\prime }$ obtained from the three types of aluminum specimens before and after corrections for attenuation and diffraction. The $\beta^{\prime }$ before all corrections appears differently according to the thickness, and it tends to increase with the thickness. Since the changes in the fundamental and second harmonic amplitudes according to the thickness change have already been reflected in the calculation of $\beta^{\prime }$, it can be thought that increasing $\beta^{\prime }$ with increasing thickness is due to the diffraction and attenuation effects. After applying all corrections, $\beta^{\prime }$ in each alloy system shows an almost constant value regardless of thickness. These results demonstrate the importance of accurate corrections in measuring relative nonlinear parameters.

When the three types of aluminum have the same thickness, the magnitude of the measured $\beta^{\prime }$ value before correction is approximately in the order of ${\beta^{\prime }}_{7075}>{\beta^{\prime }}_{6061}>{\beta^{\prime }}_{2024}$. In each type of aluminum, the $\beta^{\prime }$ value after correction is almost constant regardless of the thickness, and the magnitude of the average $\beta^{\prime }$ value is also in this order, as shown in Table 3 below. The order of magnitude of the $\beta^{\prime }$ after correction coincides with the order of magnitude of the absolute nonlinear parameter (β) after the correction shown in Table 4.

**Figure 9:**
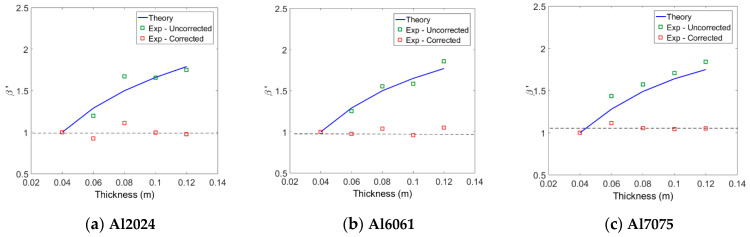
Comparison of normalized $\beta^{\prime }$ between the experiments and theory.

Next, the uncorrected $\beta^{\prime }$ values measured for each aluminum system were normalized by the $\beta^{\prime }$ of the 4 cm specimen in each system and compared with the theoretical predictions. The comparisons between the two results are shown in Figure 9, and the overall agreement is found to be pretty good. The measured $\beta^{\prime }$ for each aluminum system was corrected for attenuation and diffraction, and then normalized by the $\beta^{\prime }$ of the 4 cm specimen in each system. The results are also shown in Figure 9. As can be expected, the normalized $\beta^{\prime }$ values in each alloy system are all close to one regardless of the specimen thickness. In fact, in each alloy system, the best fit line to the five normalized data is a horizontal line passing close to one. These results validate the proposed method of measuring material nonlinear parameters using an air-coupled transducer. These results also demonstrate the importance of accurate corrections in measuring relative nonlinear parameters.

The mean, maximum, and minimum values were calculated for the corrected $\beta^{\prime }$ of each alloy system shown in Figure 8, and the results are listed in Table 3. The calculated mean $\beta^{\prime }$ for each system in Table 3 will be used as the ${\beta^{\prime }}_{\mathrm{ref}}$ of the reference specimen or the $\beta^{\prime }$ of the target specimen in the calculation of the $\beta^{\prime }/{\beta^{\prime }}_{\mathrm{ref}}$ ratio in Table 5. Table 4 shows the absolute nonlinear parameter (β) measured by the through-transmission method for the same specimens in Table 3 using a contact transmitter and a contact receiver. In this case, it is necessary to use the calibrated receiver, and detailed measurement procedures are given in Ref. [33]. The mean β value for each system in Table 4 will be used as the reference value or target value in the calculation of the $\beta /\beta_{\mathrm{ref}}$ ratio. Below, we will compare the $\beta^{\prime }/{\beta^{\prime }}_{\mathrm{ref}}$ ratio and the $\beta /\beta_{\mathrm{ref}}$ ratio.

Equation (25) indicates that the relative nonlinear parameter ratio and the absolute nonlinear parameter ratio are the same regardless of the type of reference specimen. Therefore, the equivalence of these two ratios was confirmed in the following way. Relative nonlinear parameter ratios were calculated for various alloy system combinations using the mean values of $\beta^{\prime }$ in Table 3. Similarly, absolute nonlinear parameter ratios were calculated using the mean values of β in Table 4. The results of these ratios for various combinations of specimen systems are presented in Table 5. Comparing these results, the overall agreement between the two ratios for all possible target and reference specimen combinations is within about 7%. In this study, we demonstrated the equivalence between the relative nonlinear parameter ratio and the absolute nonlinear parameter ratio using the reference samples whose acoustic impedances are not very different from the target samples. However, even if the difference in acoustic impedance between the reference sample and the target sample is large, the method can be applied in principle because only the diffraction and attenuation corrections need to be accurately calculated and applied.

Equation (26) shows that the absolute nonlinear parameter of a target specimen (β) can be obtained from the relative nonlinear parameter ratio of a target sample to a reference sample $\left(\beta^{\prime }/{\beta^{\prime }}_{\mathrm{ref}}\right)$ by multiplying the absolute nonlinear parameter of the reference specimen ($\beta_{\mathrm{ref}}$). Based on this equation, the absolute β of each aluminum type was calculated using the $\beta^{\prime }/{\beta^{\prime }}_{\mathrm{ref}}$ in Table 5 and $\beta_{\mathrm{ref}}$ in Table 4. Table 6 contains the results of this calculation, and when compared with the directly measured β of Table 4, the overall agreement is within 7%. These results show that the absolute nonlinear parameter can also be obtained within the same level of error as the absolute nonlinear parameter ratio previously observed in Table 5.

In principle, there are no restrictions on the selection of reference specimens when measuring $\beta^{\prime }/{\beta^{\prime }}_{\mathrm{ref}}$, but a standardized reference specimen is more preferable. In linear ultrasonic testing, reference standards are mainly used to to establish a general level of consistency in measurements and to calibrate instruments, and a wide variety of standard calibration blocks of different designs, sizes and system of units are available [38]. However, such reference blocks are not yet available in nonlinear ultrasonic testing. The development of standardized reference blocks related to the measurement of nonlinear parameters will be another subject of future work.

## 6. Conclusions

This paper has covered the measurement of absolute nonlinear parameters of solid specimens using an air-coupled transducer. The relative nonlinear parameter ($\beta^{\prime }$) was measured for three types of aluminum specimens with various thicknesses, and the following conclusions can be drawn:

- The ratio of the relative nonlinear parameters ($\beta^{\prime }/{\beta^{\prime }}_{\mathrm{ref}}$) and the ratio of the absolute nonlinear parameters ($\beta /\beta_{\mathrm{ref}}$) matched well within 6–7% for different target and reference specimen combinations.
- The absolute nonlinear parameter (β) obtained from the $\beta^{\prime }/{\beta^{\prime }}_{\mathrm{ref}}$ also agreed with the directly measured β.
- The proposed method does not require calibration of the air-coupled receiver, and there are no restrictions on the type and thickness of the reference specimen.
- The measurement of the β of a target specimen requires the β of the reference specimen.
- The received signal from the air-coupled transducer can be affected by the surface roughness of the specimen, alignment of the transmitter and receiver, and vibration of the experiment table.
- The use of low frequencies is relatively inefficient in second harmonic generation, and it is not easy to apply them to thin specimens.

## Figures and Tables

**Table 1:** Acoustic parameters of the two-layered medium used in the wave field calculation.

Materials	Wave Speed (m/s)	Density (kg/m^3^)	Attenuation (Np/m)
Al6061	6422	2700	$\alpha_{1}=0.5,\;\alpha_{2}=3\alpha_{1}$
Air	346	1.29	$1.83\times 10^{-11}f^{2}$

**Table 2:** Surface roughness of aluminum specimens (Unit: μm).

Materials	Specimen Thickness (cm)				
	4	6	8	10	12
Al2024	0.12	0.10	0.13	0.09	0.12
Al6061	0.11	0.09	0.16	0.10	0.10
Al7075	0.11	0.10	0.14	0.12	0.10

**Table 3:** Measurement results of the relative nonlinear parameter $\beta^{\prime }$.

Material	${\beta^{\prime }}_{\mathrm{mean}}$	${\beta^{\prime }}_{\max}$	${\beta^{\prime }}_{\min}$
Al2024	$2.39\times 10^{-4}$	$2.65\times 10^{-4}$	$2.21\times 10^{-4}$
Al6061	$2.82\times 10^{-4}$	$2.95\times 10^{-4}$	$2.70\times 10^{-4}$
Al7075	$3.22\times 10^{-4}$	$3.41\times 10^{-4}$	$3.06\times 10^{-4}$

**Table 4:** Measurement results of the absolute nonlinear parameter β.

Material	$\beta_{\mathrm{mean}}$	$\beta_{\max}$	$\beta_{\min}$
Al2024	4.78	5.13	4.66
Al6061	5.32	5.43	5.21
Al7075	6.46	6.68	6.31

**Table 5:** Comparison of the relative nonlinear parameter ratio and absolute nonlinear parameter ratio.

Ratio	$\beta^{\prime }/{\beta^{\prime }}_{\mathrm{ref}}$	$\beta /\beta_{\mathrm{ref}}$	Difference (%)
Al6061/Al2024	1.18	1.11	6.3
Al7075/Al2024	1.35	1.35	0
Al2024/Al6061	0.85	0.90	5.6
Al7075/Al6061	1.14	1.21	5.8
Al2024/Al7075	0.74	0.74	0
Al6061/Al7075	0.88	0.82	7.3

**Table 6:** Absolute nonlinear parameter obtained from the relative nonlinear parameter ratio of two materials.

Ratio	$\beta^{\prime }/{\beta^{\prime }}_{\mathrm{ref}}$	$\frac{\beta^{\prime }}{{\beta^{\prime }}_{\mathrm{ref}}}\times \beta_{\mathrm{ref}}=\beta$	Difference (%)
Al2024/Al6061	0.85	4.52	5.7
Al2024/Al7075	0.74	4.78	0
Al6061/Al2024	1.18	5.64	6.0
Al6061/Al7075	0.88	5.68	6.8
Al7075/Al2024	1.35	6.45	0.2
Al7075/Al6061	1.14	6.06	6.6

## Data Availability

The data presented in this study are available on request from the corresponding author.

## Nomenclature

a	Transmitter radius
b	Receiver radius
A(ω)	Magnitude spectrum
$\alpha_{n}^{\left(m\right)}$	Attenuation coefficient of *n*th harmonic in *m*th layer
β	Absolute nonlinear parameter
$\beta^{\prime }$	Relative nonlinear parameter
$\beta_{\mathrm{ref}}$	Absolute nonlinear parameter of reference specimen
${\beta^{\prime }}_{\mathrm{ref}}$	Relative nonlinear parameter of reference specimen
$c^{\left(m\right)}$	Longitudinal wave velocity in *m*th layer
$C_{11}$	Second order stiffness component
$C_{111}$	Third order stiffness component
$D_{n}^{\left(m\right)}$	Diffraction correction of *n*th harmonic in *m*th layer
f	Frequency
$G_{n}^{\left(m\right)}$	Green function of *n*th harmonic in *m*th layer
H(ω)	Transfer function of receiving transducer
$k_{n}^{\left(m\right)}$	Wave number of *n*th harmonic in *m*th layer
$M_{n}^{\left(m\right)}$	Attenuation correction of *n*th harmonic in *m*th layer
$r_{n}$	Distance from source to target point for *n*th harmonic
$T_{12}$	Transmission coefficient at the interface of medium 1 and medium 2
$u_{n}^{\left(m\right)}$	Displacement of *n*th harmonic in *m*th layer
${\tilde{u}}_{n}^{\left(m\right)}$	Received average displacement of *n*th harmonic in *m*th layer
$U_{n}^{\left(m\right)}$	Displacement amplitude of *n*th harmonic in *m*th layer
$U_{0}$	Source displacement
$z_{m}$	Thickness of *m*th layer
$Z_{m}$	Acoustic impedence of *m*th layer
(x,y,z)	Cartesian coordinates
